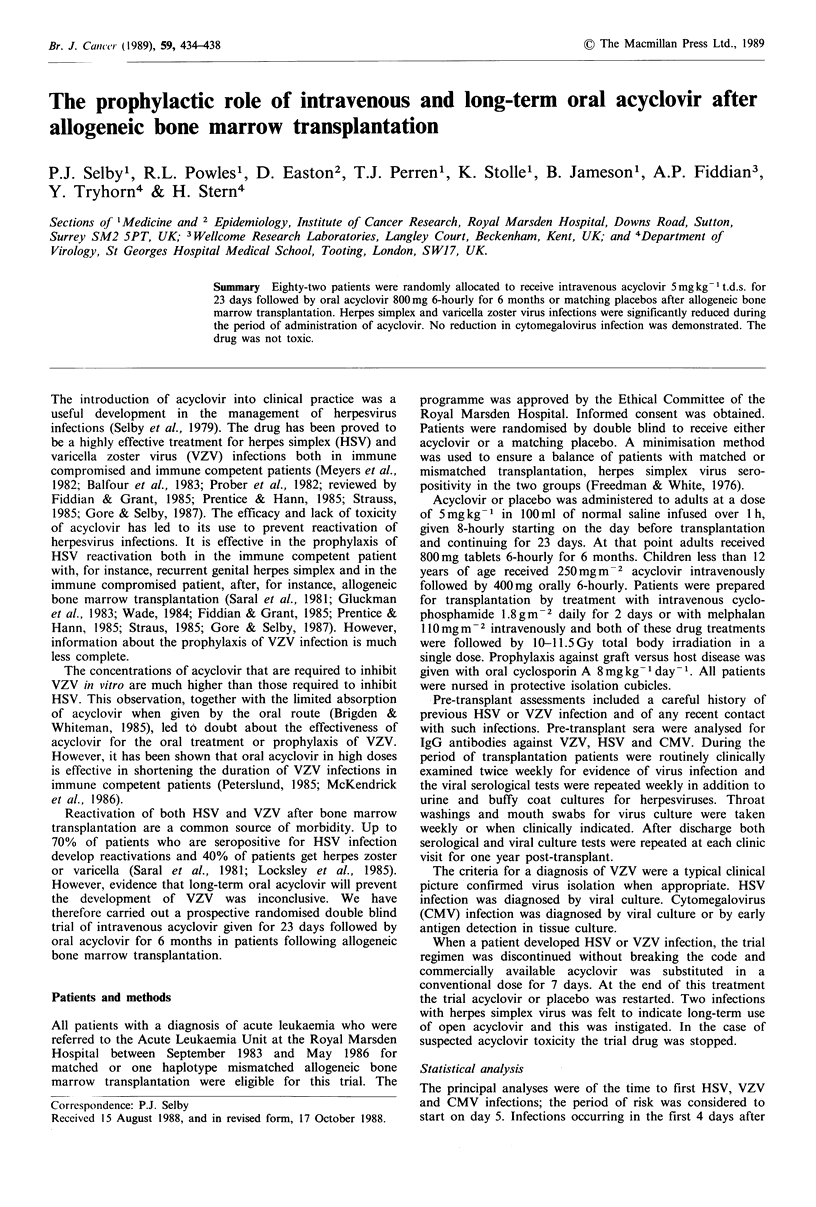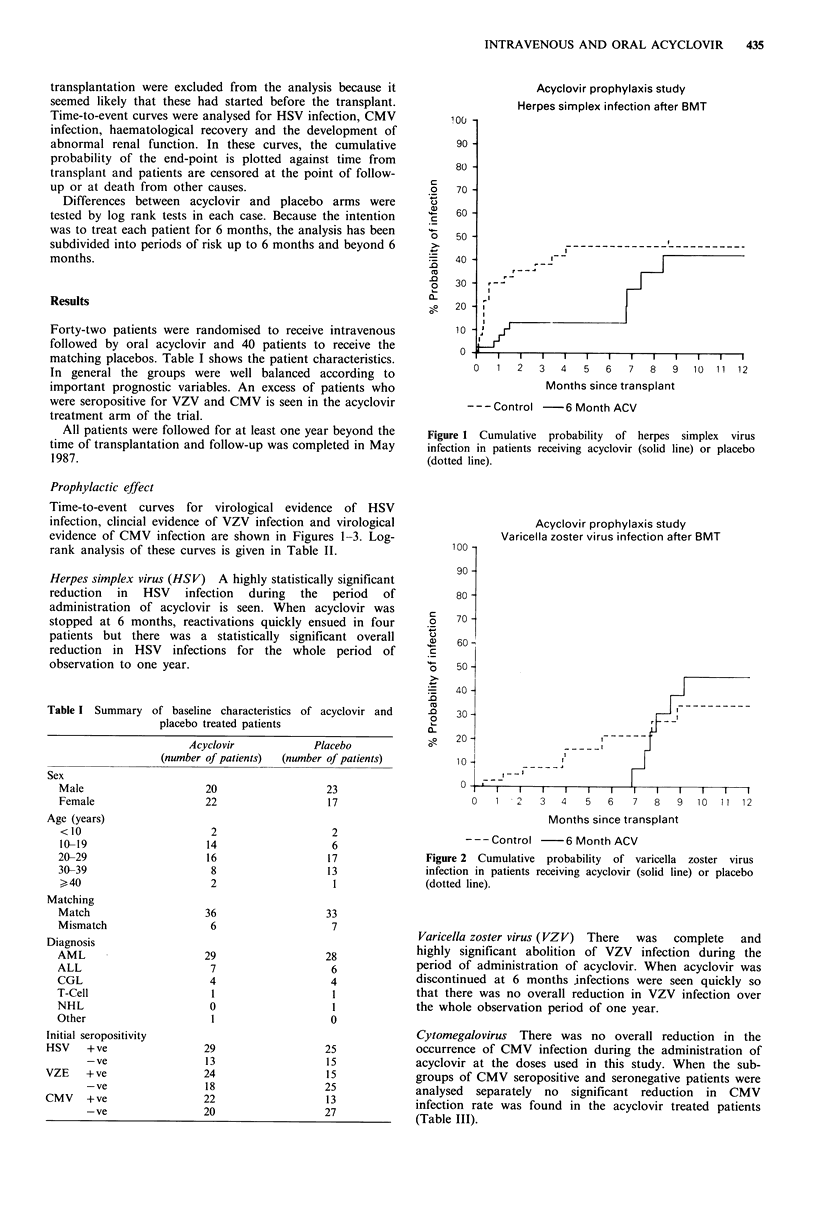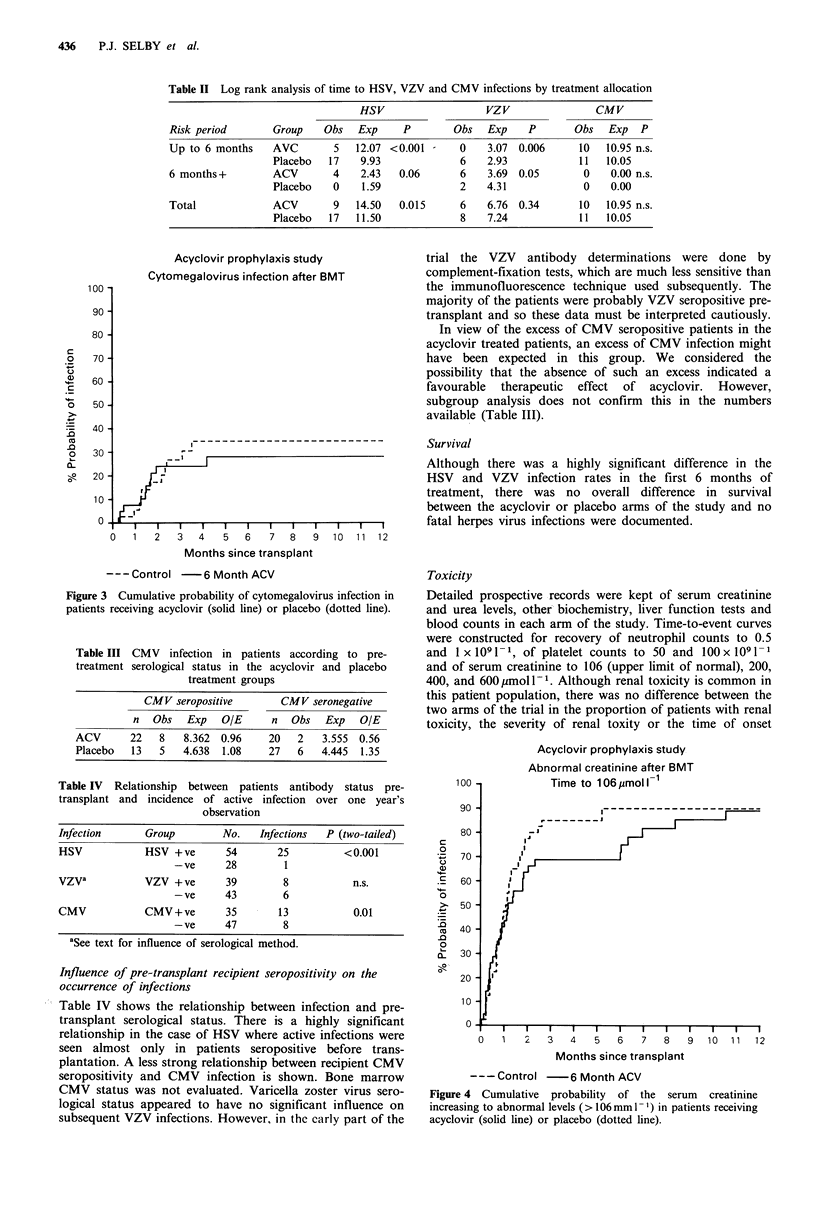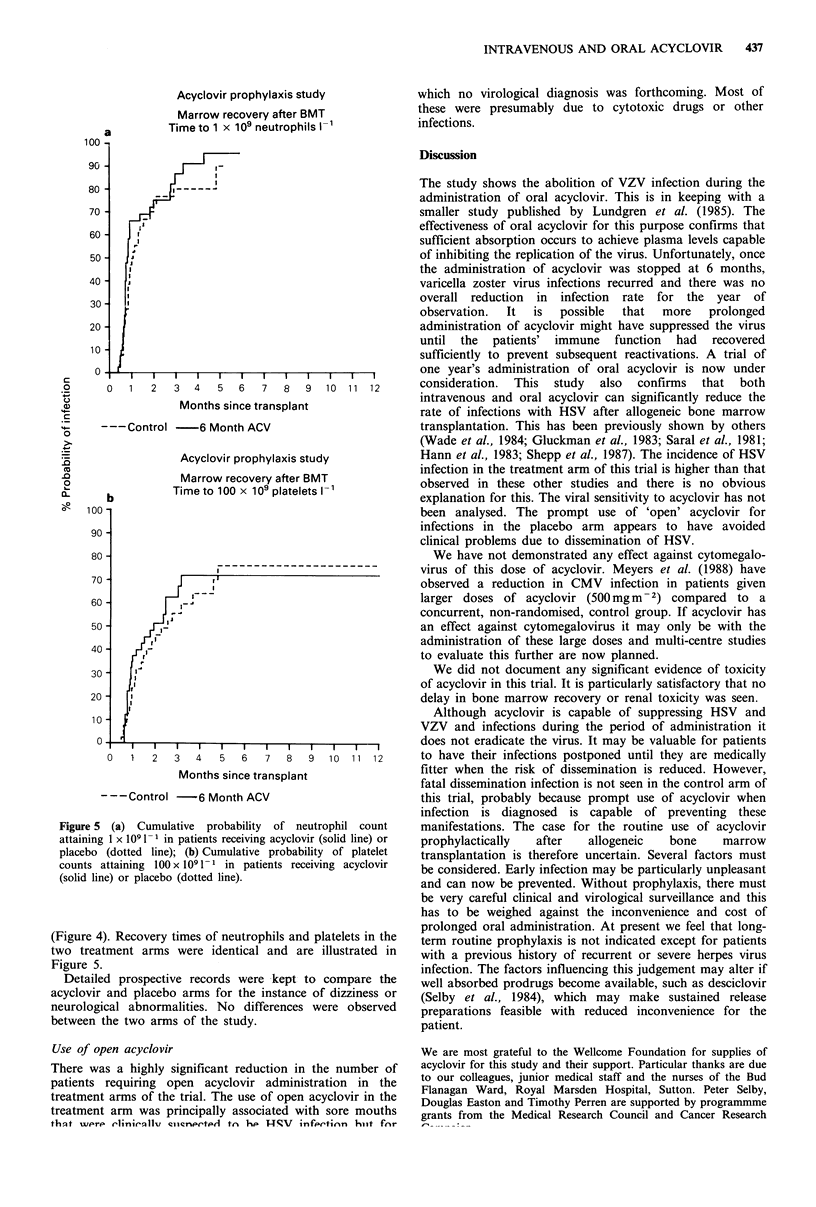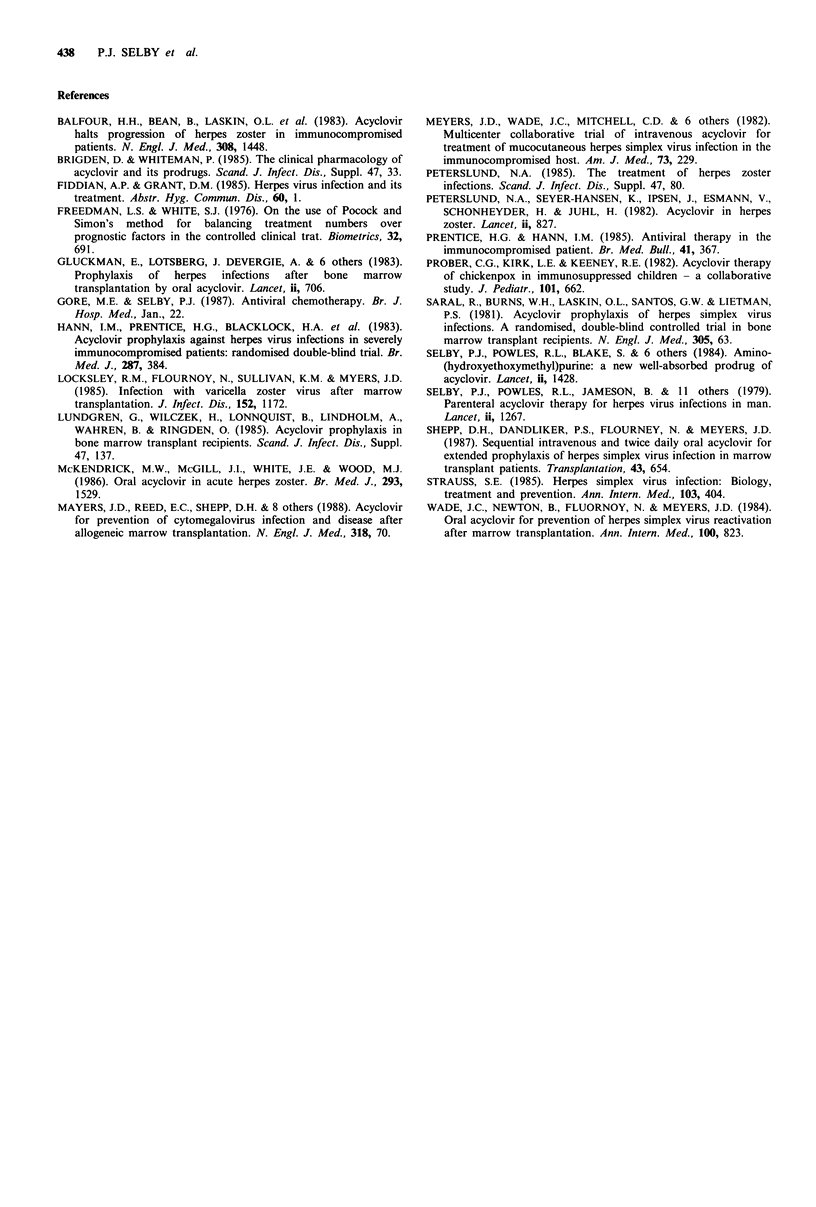# The prophylactic role of intravenous and long-term oral acyclovir after allogeneic bone marrow transplantation.

**DOI:** 10.1038/bjc.1989.88

**Published:** 1989-03

**Authors:** P. J. Selby, R. L. Powles, D. Easton, T. J. Perren, K. Stolle, B. Jameson, A. P. Fiddian, Y. Tryhorn, H. Stern

**Affiliations:** Institute of Cancer Research, Royal Marsden Hospital, Surrey, UK.

## Abstract

Eighty-two patients were randomly allocated to receive intravenous acyclovir 5 mg kg-1 t.d.s. for 23 days followed by oral acyclovir 800 mg 6-hourly for 6 months or matching placebos after allogeneic bone marrow transplantation. Herpes simplex and varicella zoster virus infections were significantly reduced during the period of administration of acyclovir. No reduction in cytomegalovirus infection was demonstrated. The drug was not toxic.


*Br. J. Cancer* (1989), **59**, 434–438 © The Macmillan Press Ltd., 1989

### The prophylactic role of intravenous and long-term oral acyclovir after allogeneic bone marrow transplantation

P.J. Selby[1], R.L. Powles[1], D. Easton[2], T.J. Perren[1], K. Stolle[1], B. Jameson[1], A.P. Fiddian[3], Y. Tryhorn[4] & H. Stern[4]

*Sections of [1]Medicine and [2] Epidemiology, Institute of Cancer Research, Royal Marsden Hospital, Downs Road, Sutton, Surrey SM2 5PT, UK; [3]Wellcome Research Laboratories, Langley Court, Beckenham, Kent, UK; and [4]Department of Virology, St Georges Hospital Medical School, Tooting, London, SW17, UK.*

**Summary** Eighty-two patients were randomly allocated to receive intravenous acyclovir $5\,mg\,kg^{-1}$ t.d.s. for 23 days followed by oral acyclovir 800 mg 6-hourly for 6 months or matching placebos after allogeneic bone marrow transplantation. Herpes simplex and varicella zoster virus infections were significantly reduced during the period of administration of acyclovir. No reduction in cytomegalovirus infection was demonstrated. The drug was not toxic.

The introduction of acyclovir into clinical practice was a useful development in the management of herpesvirus infections (Selby *et al.*, 1979). The drug has been proved to be a highly effective treatment for herpes simplex (HSV) and varicella zoster virus (VZV) infections both in immune compromised and immune competent patients (Meyers *et al.*, 1982; Balfour *et al.*, 1983; Prober *et al.*, 1982; reviewed by Fiddian & Grant, 1985; Prentice & Hann, 1985; Strauss, 1985; Gore & Selby, 1987). The efficacy and lack of toxicity of acyclovir has led to its use to prevent reactivation of herpesvirus infections. It is effective in the prophylaxis of HSV reactivation both in the immune competent patient with, for instance, recurrent genital herpes simplex and in the immune compromised patient, after, for instance, allogeneic bone marrow transplantation (Saral *et al.*, 1981; Gluckman *et al.*, 1983; Wade, 1984; Fiddian & Grant, 1985; Prentice & Hann, 1985; Straus, 1985; Gore & Selby, 1987). However, information about the prophylaxis of VZV infection is much less complete.

The concentrations of acyclovir that are required to inhibit VZV *in vitro* are much higher than those required to inhibit HSV. This observation, together with the limited absorption of acyclovir when given by the oral route (Brigden & Whiteman, 1985), led to doubt about the effectiveness of acyclovir for the oral treatment or prophylaxis of VZV. However, it has been shown that oral acyclovir in high doses is effective in shortening the duration of VZV infections in immune competent patients (Peterslund, 1985; McKendrick *et al.*, 1986).

Reactivation of both HSV and VZV after bone marrow transplantation are a common source of morbidity. Up to 70% of patients who are seropositive for HSV infection develop reactivations and 40% of patients get herpes zoster or varicella (Saral *et al.*, 1981; Locksley *et al.*, 1985). However, evidence that long-term oral acyclovir will prevent the development of VZV was inconclusive. We have therefore carried out a prospective randomised double blind trial of intravenous acyclovir given for 23 days followed by oral acyclovir for 6 months in patients following allogeneic bone marrow transplantation.

### Patients and methods

All patients with a diagnosis of acute leukaemia who were referred to the Acute Leukaemia Unit at the Royal Marsden Hospital between September 1983 and May 1986 for matched or one haplotype mismatched allogeneic bone marrow transplantation were eligible for this trial. The programme was approved by the Ethical Committee of the Royal Marsden Hospital. Informed consent was obtained. Patients were randomised by double blind to receive either acyclovir or a matching placebo. A minimisation method was used to ensure a balance of patients with matched or mismatched transplantation, herpes simplex virus seropositivity in the two groups (Freedman & White, 1976).

Acyclovir or placebo was administered to adults at a dose of $5\,mg\,kg^{-1}$ in 100 ml of normal saline infused over 1 h, given 8-hourly starting on the day before transplantation and continuing for 23 days. At that point adults received 800 mg tablets 6-hourly for 6 months. Children less than 12 years of age received $250\,mg\,m^{-2}$ acyclovir intravenously followed by 400 mg orally 6-hourly. Patients were prepared for transplantation by treatment with intravenous cyclophosphamide $1.8\,g\,m^{-2}$ daily for 2 days or with melphalan $110\,mg\,m^{-2}$ intravenously and both of these drug treatments were followed by 10–11.5 Gy total body irradiation in a single dose. Prophylaxis against graft versus host disease was given with oral cyclosporin A $8\,mg\,kg^{-1}\,day^{-1}$. All patients were nursed in protective isolation cubicles.

Pre-transplant assessments included a careful history of previous HSV or VZV infection and of any recent contact with such infections. Pre-transplant sera were analysed for IgG antibodies against VZV, HSV and CMV. During the period of transplantation patients were routinely clinically examined twice weekly for evidence of virus infection and the viral serological tests were repeated weekly in addition to urine and buffy coat cultures for herpesviruses. Throat washings and mouth swabs for virus culture were taken weekly or when clinically indicated. After discharge both serological and viral culture tests were repeated at each clinic visit for one year post-transplant.

The criteria for a diagnosis of VZV were a typical clinical picture confirmed virus isolation when appropriate. HSV infection was diagnosed by viral culture. Cytomegalovirus (CMV) infection was diagnosed by viral culture or by early antigen detection in tissue culture.

When a patient developed HSV or VZV infection, the trial regimen was discontinued without breaking the code and commercially available acyclovir was substituted in a conventional dose for 7 days. At the end of this treatment the trial acyclovir or placebo was restarted. Two infections with herpes simplex virus was felt to indicate long-term use of open acyclovir and this was instigated. In the case of suspected acyclovir toxicity the trial drug was stopped.

### *Statistical analysis*

The principal analyses were of the time to first HSV, VZV and CMV infections; the period of risk was considered to start on day 5. Infections occurring in the first 4 days after

Correspondence: P.J. Selby
Received 15 August 1988, and in revised form, 17 October 1988.

transplantation were excluded from the analysis because it seemed likely that these had started before the transplant. Time-to-event curves were analysed for HSV infection, CMV infection, haematological recovery and the development of abnormal renal function. In these curves, the cumulative probability of the end-point is plotted against time from transplant and patients are censored at the point of follow-up or at death from other causes.

Differences between acyclovir and placebo arms were tested by log rank tests in each case. Because the intention was to treat each patient for 6 months, the analysis has been subdivided into periods of risk up to 6 months and beyond 6 months.

### Results

Forty-two patients were randomised to receive intravenous followed by oral acyclovir and 40 patients to receive the matching placebos. Table I shows the patient characteristics. In general the groups were well balanced according to important prognostic variables. An excess of patients who were seropositive for VZV and CMV is seen in the acyclovir treatment arm of the trial.

All patients were followed for at least one year beyond the time of transplantation and follow-up was completed in May 1987.

### *Prophylactic effect*

Time-to-event curves for virological evidence of HSV infection, clincial evidence of VZV infection and virological evidence of CMV infection are shown in Figures 1–3. Log-rank analysis of these curves is given in Table II.

*Herpes simplex virus* (*HSV*) A highly statistically significant reduction in HSV infection during the period of administration of acyclovir is seen. When acyclovir was stopped at 6 months, reactivations quickly ensued in four patients but there was a statistically significant overall reduction in HSV infections for the whole period of observation to one year.

**Table I** Summary of baseline characteristics of acyclovir and placebo treated patients

| | *Acyclovir* (*number of patients*) | *Placebo* (*number of patients*) |
|---|---|---|
| Sex | | |
| Male | 20 | 23 |
| Female | 22 | 17 |
| Age (years) | | |
| <10 | 2 | 2 |
| 10–19 | 14 | 6 |
| 20–29 | 16 | 17 |
| 30–39 | 8 | 13 |
| ⩾40 | 2 | 1 |
| Matching | | |
| Match | 36 | 33 |
| Mismatch | 6 | 7 |
| Diagnosis | | |
| AML | 29 | 28 |
| ALL | 7 | 6 |
| CGL | 4 | 4 |
| T-Cell | 1 | 1 |
| NHL | 0 | 1 |
| Other | 1 | 0 |
| Initial seropositivity | | |
| HSV +ve | 29 | 25 |
| −ve | 13 | 15 |
| VZE +ve | 24 | 15 |
| −ve | 18 | 25 |
| CMV +ve | 22 | 13 |
| −ve | 20 | 27 |

**Figure 1** Cumulative probability of herpes simplex virus infection in patients receiving acyclovir (solid line) or placebo (dotted line).

**Figure 2** Cumulative probability of varicella zoster virus infection in patients receiving acyclovir (solid line) or placebo (dotted line).

*Varicella zoster virus* (*VZV*) There was complete and highly significant abolition of VZV infection during the period of administration of acyclovir. When acyclovir was discontinued at 6 months infections were seen quickly so that there was no overall reduction in VZV infection over the whole observation period of one year.

*Cytomegalovirus* There was no overall reduction in the occurrence of CMV infection during the administration of acyclovir at the doses used in this study. When the subgroups of CMV seropositive and seronegative patients were analysed separately no significant reduction in CMV infection rate was found in the acyclovir treated patients (Table III).

**Table II** Log rank analysis of time to HSV, VZV and CMV infections by treatment allocation

| | | *HSV* | | | *VZV* | | | *CMV* | | |
|---|---|---|---|---|---|---|---|---|---|---|
| *Risk period* | *Group* | *Obs* | *Exp* | *P* | *Obs* | *Exp* | *P* | *Obs* | *Exp* | *P* |
| Up to 6 months | AVC | 5 | 12.07 | <0.001 | 0 | 3.07 | 0.006 | 10 | 10.95 | n.s. |
| | Placebo | 17 | 9.93 | | 6 | 2.93 | | 11 | 10.05 | |
| 6 months + | ACV | 4 | 2.43 | 0.06 | 6 | 3.69 | 0.05 | 0 | 0.00 | n.s. |
| | Placebo | 0 | 1.59 | | 2 | 4.31 | | 0 | 0.00 | |
| Total | ACV | 9 | 14.50 | 0.015 | 6 | 6.76 | 0.34 | 10 | 10.95 | n.s. |
| | Placebo | 17 | 11.50 | | 8 | 7.24 | | 11 | 10.05 | |

**Figure 3** Cumulative probability of cytomegalovirus infection in patients receiving acyclovir (solid line) or placebo (dotted line).

**Table III** CMV infection in patients according to pre-treatment serological status in the acyclovir and placebo treatment groups

| | *CMV seropositive* | | | | *CMV seronegative* | | | |
|---|---|---|---|---|---|---|---|---|
| | *n* | *Obs* | *Exp* | *O/E* | *n* | *Obs* | *Exp* | *O/E* |
| ACV | 22 | 8 | 8.362 | 0.96 | 20 | 2 | 3.555 | 0.56 |
| Placebo | 13 | 5 | 4.638 | 1.08 | 27 | 6 | 4.445 | 1.35 |

**Table IV** Relationship between patients antibody status pre-transplant and incidence of active infection over one year's observation

| *Infection* | *Group* | *No.* | *Infections* | *P (two-tailed)* |
|---|---|---|---|---|
| HSV | HSV +ve | 54 | 25 | <0.001 |
| | −ve | 28 | 1 | |
| VZV[a] | VZV +ve | 39 | 8 | n.s. |
| | −ve | 43 | 6 | |
| CMV | CMV+ve | 35 | 13 | 0.01 |
| | −ve | 47 | 8 | |

[a]See text for influence of serological method.

*Influence of pre-transplant recipient seropositivity on the occurrence of infections*

Table IV shows the relationship between infection and pre-transplant serological status. There is a highly significant relationship in the case of HSV where active infections were seen almost only in patients seropositive before transplantation. A less strong relationship between recipient CMV seropositivity and CMV infection is shown. Bone marrow CMV status was not evaluated. Varicella zoster virus serological status appeared to have no significant influence on subsequent VZV infections. However, in the early part of the trial the VZV antibody determinations were done by complement-fixation tests, which are much less sensitive than the immunofluorescence technique used subsequently. The majority of the patients were probably VZV seropositive pre-transplant and so these data must be interpreted cautiously.

In view of the excess of CMV seropositive patients in the acyclovir treated patients, an excess of CMV infection might have been expected in this group. We considered the possibility that the absence of such an excess indicated a favourable therapeutic effect of acyclovir. However, subgroup analysis does not confirm this in the numbers available (Table III).

*Survival*

Although there was a highly significant difference in the HSV and VZV infection rates in the first 6 months of treatment, there was no overall difference in survival between the acyclovir or placebo arms of the study and no fatal herpes virus infections were documented.

*Toxicity*

Detailed prospective records were kept of serum creatinine and urea levels, other biochemistry, liver function tests and blood counts in each arm of the study. Time-to-event curves were constructed for recovery of neutrophil counts to 0.5 and $1 \times 10^9 l^{-1}$, of platelet counts to 50 and $100 \times 10^9 l^{-1}$ and of serum creatinine to 106 (upper limit of normal), 200, 400, and $600 \mu mol l^{-1}$. Although renal toxicity is common in this patient population, there was no difference between the two arms of the trial in the proportion of patients with renal toxicity, the severity of renal toxity or the time of onset

**Figure 4** Cumulative probability of the serum creatinine increasing to abnormal levels ($>106 mm l^{-1}$) in patients receiving acyclovir (solid line) or placebo (dotted line).

**Figure 5** (**a**) Cumulative probability of neutrophil count attaining $1 \times 10^9 l^{-1}$ in patients receiving acyclovir (solid line) or placebo (dotted line); (**b**) Cumulative probability of platelet counts attaining $100 \times 10^9 l^{-1}$ in patients receiving acyclovir (solid line) or placebo (dotted line).

(Figure 4). Recovery times of neutrophils and platelets in the two treatment arms were identical and are illustrated in Figure 5.

Detailed prospective records were kept to compare the acyclovir and placebo arms for the instance of dizziness or neurological abnormalities. No differences were observed between the two arms of the study.

*Use of open acyclovir*

There was a highly significant reduction in the number of patients requiring open acyclovir administration in the treatment arms of the trial. The use of open acyclovir in the treatment arm was principally associated with sore mouths that were clinically suspected to be HSV infection but for which no virological diagnosis was forthcoming. Most of these were presumably due to cytotoxic drugs or other infections.

### Discussion

The study shows the abolition of VZV infection during the administration of oral acyclovir. This is in keeping with a smaller study published by Lundgren *et al.* (1985). The effectiveness of oral acyclovir for this purpose confirms that sufficient absorption occurs to achieve plasma levels capable of inhibiting the replication of the virus. Unfortunately, once the administration of acyclovir was stopped at 6 months, varicella zoster virus infections recurred and there was no overall reduction in infection rate for the year of observation. It is possible that more prolonged administration of acyclovir might have suppressed the virus until the patients' immune function had recovered sufficiently to prevent subsequent reactivations. A trial of one year's administration of oral acyclovir is now under consideration. This study also confirms that both intravenous and oral acyclovir can significantly reduce the rate of infections with HSV after allogeneic bone marrow transplantation. This has been previously shown by others (Wade *et al.*, 1984; Gluckman *et al.*, 1983; Saral *et al.*, 1981; Hann *et al.*, 1983; Shepp *et al.*, 1987). The incidence of HSV infection in the treatment arm of this trial is higher than that observed in these other studies and there is no obvious explanation for this. The viral sensitivity to acyclovir has not been analysed. The prompt use of 'open' acyclovir for infections in the placebo arm appears to have avoided clinical problems due to dissemination of HSV.

We have not demonstrated any effect against cytomegalovirus of this dose of acyclovir. Meyers *et al.* (1988) have observed a reduction in CMV infection in patients given larger doses of acyclovir ($500 \text{mg} \text{m}^{-2}$) compared to a concurrent, non-randomised, control group. If acyclovir has an effect against cytomegalovirus it may only be with the administration of these large doses and multi-centre studies to evaluate this further are now planned.

We did not document any significant evidence of toxicity of acyclovir in this trial. It is particularly satisfactory that no delay in bone marrow recovery or renal toxicity was seen.

Although acyclovir is capable of suppressing HSV and VZV and infections during the period of administration it does not eradicate the virus. It may be valuable for patients to have their infections postponed until they are medically fitter when the risk of dissemination is reduced. However, fatal dissemination infection is not seen in the control arm of this trial, probably because prompt use of acyclovir when infection is diagnosed is capable of preventing these manifestations. The case for the routine use of acyclovir prophylactically after allogeneic bone marrow transplantation is therefore uncertain. Several factors must be considered. Early infection may be particularly unpleasant and can now be prevented. Without prophylaxis, there must be very careful clinical and virological surveillance and this has to be weighed against the inconvenience and cost of prolonged oral administration. At present we feel that long-term routine prophylaxis is not indicated except for patients with a previous history of recurrent or severe herpes virus infection. The factors influencing this judgement may alter if well absorbed prodrugs become available, such as desciclovir (Selby *et al.*, 1984), which may make sustained release preparations feasible with reduced inconvenience for the patient.

We are most grateful to the Wellcome Foundation for supplies of acyclovir for this study and their support. Particular thanks are due to our colleagues, junior medical staff and the nurses of the Bud Flanagan Ward, Royal Marsden Hospital, Sutton. Peter Selby, Douglas Easton and Timothy Perren are supported by programmme grants from the Medical Research Council and Cancer Research [illegible]

### References

BALFOUR, H.H., BEAN, B., LASKIN, O.L. *et al.* (1983). Acyclovir halts progression of herpes zoster in immunocompromised patients. *N. Engl. J. Med.*, **308**, 1448.

BRIGDEN, D. & WHITEMAN, P. (1985). The clinical pharmacology of acyclovir and its prodrugs. *Scand. J. Infect. Dis.*, Suppl. 47, 33.

FIDDIAN, A.P. & GRANT, D.M. (1985). Herpes virus infection and its treatment. *Abstr. Hyg. Commun. Dis.*, **60**, 1.

FREEDMAN, L.S. & WHITE, S.J. (1976). On the use of Pocock and Simon's method for balancing treatment numbers over prognostic factors in the controlled clinical trat. *Biometrics*, **32**, 691.

GLUCKMAN, E., LOTSBERG, J. DEVERGIE, A. & 6 others (1983). Prophylaxis of herpes infections after bone marrow transplantation by oral acyclovir. *Lancet*, **ii**, 706.

GORE, M.E. & SELBY, P.J. (1987). Antiviral chemotherapy. *Br. J. Hosp. Med.*, Jan., 22.

HANN, I.M., PRENTICE, H.G., BLACKLOCK, H.A. *et al.* (1983). Acyclovir prophylaxis against herpes virus infections in severely immunocompromised patients: randomised double-blind trial. *Br. Med. J.*, **287**, 384.

LOCKSLEY, R.M., FLOURNOY, N., SULLIVAN, K.M. & MYERS, J.D. (1985). Infection with varicella zoster virus after marrow transplantation. *J. Infect. Dis.*, **152**, 1172.

LUNDGREN, G., WILCZEK, H., LONNQUIST, B., LINDHOLM, A., WAHREN, B. & RINGDEN, O. (1985). Acyclovir prophylaxis in bone marrow transplant recipients. *Scand. J. Infect. Dis.*, Suppl. 47, 137.

McKENDRICK, M.W., McGILL, J.I., WHITE, J.E. & WOOD, M.J. (1986). Oral acyclovir in acute herpes zoster. *Br. Med. J.*, **293**, 1529.

MAYERS, J.D., REED, E.C., SHEPP, D.H. & 8 others (1988). Acyclovir for prevention of cytomegalovirus infection and disease after allogeneic marrow transplantation. *N. Engl. J. Med.*, **318**, 70.

MEYERS, J.D., WADE, J.C., MITCHELL, C.D. & 6 others (1982). Multicenter collaborative trial of intravenous acyclovir for treatment of mucocutaneous herpes simplex virus infection in the immunocompromised host. *Am. J. Med.*, **73**, 229.

PETERSLUND, N.A. (1985). The treatment of herpes zoster infections. *Scand. J. Infect. Dis.*, Suppl. 47, 80.

PETERSLUND, N.A., SEYER-HANSEN, K., IPSEN, J., ESMANN, V., SCHONHEYDER, H. & JUHL, H. (1982). Acyclovir in herpes zoster. *Lancet*, **ii**, 827.

PRENTICE, H.G. & HANN, I.M. (1985). Antiviral therapy in the immunocompromised patient. *Br. Med. Bull.*, **41**, 367.

PROBER, C.G., KIRK, L.E. & KEENEY, R.E. (1982). Acyclovir therapy of chickenpox in immunosuppressed children – a collaborative study. *J. Pediatr.*, **101**, 662.

SARAL, R., BURNS, W.H., LASKIN, O.L., SANTOS, G.W. & LIETMAN, P.S. (1981). Acyclovir prophylaxis of herpes simplex virus infections. A randomised, double-blind controlled trial in bone marrow transplant recipients. *N. Engl. J. Med.*, **305**, 63.

SELBY, P.J., POWLES, R.L., BLAKE, S. & 6 others (1984). Amino-(hydroxyethoxymethyl)purine: a new well-absorbed prodrug of acyclovir. *Lancet*, **ii**, 1428.

SELBY, P.J., POWLES, R.L., JAMESON, B. & 11 others (1979). Parenteral acyclovir therapy for herpes virus infections in man. *Lancet*, **ii**, 1267.

SHEPP, D.H., DANDLIKER, P.S., FLOURNEY, N. & MEYERS, J.D. (1987). Sequential intravenous and twice daily oral acyclovir for extended prophylaxis of herpes simplex virus infection in marrow transplant patients. *Transplantation*, **43**, 654.

STRAUSS, S.E. (1985). Herpes simplex virus infection: Biology, treatment and prevention. *Ann. Intern. Med.*, **103**, 404.

WADE, J.C., NEWTON, B., FLUORNOY, N. & MEYERS, J.D. (1984). Oral acyclovir for prevention of herpes simplex virus reactivation after marrow transplantation. *Ann. Intern. Med.*, **100**, 823.